# Stability After Legal Gender Change Among Adults With Gender Dysphoria

**DOI:** 10.1001/jamanetworkopen.2025.27780

**Published:** 2025-09-04

**Authors:** Kristen D. Clark, Richard A. White, Georgios Karamanis, Malin Indremo, Fatih Özel, Alkistis Skalkidou, Thomas Frisell, Fotios C. Papadopoulos

**Affiliations:** 1 Department of Medical Sciences, Clinical Psychiatry, Uppsala University, Uppsala, Sweden; 2 Norwegian Institute of Public Health, Oslo, Norway; 3 Department of Psychiatry, Uppsala University Hospital, Uppsala, Sweden; 4 Department of Organismal Biology, Uppsala University, Uppsala, Sweden; 5 Centre for Women’s Mental Health During the Reproductive Lifespan–Womher, Uppsala University, Uppsala, Sweden; 6 Department of Women’s and Children’s Health, Uppsala University, Uppsala, Sweden; 7 Clinical Epidemiology Division, Department of Medicine Solna, Karolinska Institutet, Stockholm, Sweden

## Abstract

**Importance:**

The number of people seeking treatment for gender dysphoria (GD) has risen in recent decades, yet data remain limited for understanding cases of detransition (ie, the reversal of social, medical, or legal transition). Legal gender change in national registers poses an opportunity to examine legal gender reversals, which may reflect an aspect of the detransition process, while also informing an understanding of the stability after a legal gender change.

**Objective:**

To determine the probability of obtaining a legal gender change and its stability over time among people diagnosed with GD in Sweden during a 10-year study period.

**Design, Setting, and Participants:**

In this population cohort study using data from Swedish national registers from January 1, 2013, to December 31, 2023, individuals who received their first *International Statistical Classification of Diseases and Related Health Problems, Tenth Revision* (*ICD-10*), diagnosis code for GD during the study period were included. While participants may have received a GD *ICD-10* code as youths, legal gender changes only occurred for adults (aged ≥18 years) based on legal requirements during the time of data collection.

**Exposure:**

Gender dysphoria.

**Main Outcomes and Measures:**

Legal gender change and its stability, that is, absence of legal gender reversal, as reported in the registry. Kaplan-Meier survival analysis was used to determine the probability of legal gender change and its stability during a 10-year study period.

**Results:**

A total of 7293 people with a GD diagnosis were included (36 025 person-years). Approximately one-third of the sample (n = 2467) obtained a legal gender change during the study period. The probability of legal gender change at 10 years following diagnosis was 58.3% (95% CI, 56.2%-60.4%). Twenty-one cases of legal gender reversal were identified. The probability of legal gender stability at 10 years following the initial change was 97.7% (95% CI, 96.3%-99.2%), with no differences by sex assigned at birth for either outcome.

**Conclusions and Relevance:**

In this cohort study using national population registry data, a significant proportion of participants with GD in Sweden obtained legal gender change within 10 years of follow-up. Despite significant growth of the population with GD, the probability of legal gender stability during the 10-year study period was high, at almost 98%. These results highlight that legal gender reversal was an infrequent occurrence in a national sample.

## Key Points

**Question:**

What is the probability that the changed legal gender among people with gender dysphoria (GD) will remain stable over time?

**Findings:**

In this national cohort study, 7293 people with GD had a 58% probability of obtaining a legal gender change over time and an almost 98% probability that the legal gender remained stable. No differences based on sex assigned at birth or age were observed.

**Meaning:**

These findings suggest that among the most recent cohort of people who have sought treatment for GD and legally changed their gender, there is a low risk for legal gender reversal.

## Teaser

This cohort study identifies the probability of obtaining an initial legal gender change and its stability (ie, absence of legal gender reversal) in a recent cohort of people diagnosed with gender dysphoria in Sweden.

## Introduction

During the past decade, there has been a notable increase in individuals with gender dysphoria (GD) seeking medical treatment.^1,2^ GD is characterized by discomfort in the alignment between one’s sex assigned at birth and gender that presents in a range of severity by transgender and nonbinary (TNB) people. Some people with GD may address this discomfort through a social transition, such as changing one’s clothing, pronouns, or hair; a medical transition using gender-affirming treatments, such as hormones or surgical procedures to modify one’s body; or a combination of both approaches. Individuals with GD may also seek to change legal documentation based on their sex assigned at birth to a gender that most closely aligns with their identity or gender expression. Concurrent with these rising GD treatment rates, concerns have emerged about the occurrence of detransition (stopping or reversing part, or all, of one’s social, medical, or legal transition), particularly in regard to adolescents and young adults assigned female sex at birth (AFAB),^3,4,5,6^ who represent a growing subgroup of people with GD seeking care.^1,7,8^ Concerns also extend to individuals whose GD symptoms emerged during adolescence or adulthood or that co-occur with mental health or neurodevelopmental conditions,^4,9^ resulting in some clinicians suggesting a need for longer or more cautious assessments.^10,11^ These debates are amplified by questions over whether there are epidemiological shifts in TNB populations that could impede the generalizability of historical research on the effectiveness of current models of gender-affirming treatment to current cohorts.^3,12^

Decisions to detransition have been attributed to a diverse set of considerations, including experiencing stigma, mistreatment, loss of family or loved ones due to one’s gender identity, a lack of resolution of GD, co-occurring mental health symptoms, internalized heterosexism, or no longer identifying as TNB.^4,13,14^ This area of research is emergent and has developed through the use of qualitative studies to identify themes across the experiences of individuals who have detransitioned and cross-sectional convenience samples.^3,14^ A recent meta-analysis^12^ found that in 1.2% of cases, patients regretted receiving a gender-affirming surgical procedure, while another systematic review on adolescents^15^ found 1.6% to 9.8% discontinued hormonal treatment, although with considerable variability in the measurement of detransition or desistence across studies. In one study using a nonclinical convenience sample,^16^ among the 16.4% of participants who reported discontinuing or reversing a gender-affirming medical treatment, approximately one-third reported doing so because of a change in their gender identity, although most continued to identify as TNB. More recent studies have sought to describe a more permanent seemingly rare type of detransition experience by surveying individuals who no longer identify as TNB, yet this has proven difficult to conceptualize and measure. For example, a recent study of individuals who have detransitioned and no longer identify as TNB^8^ found that one-third of participants reported that they may again identify as TNB at some point in the future. The timeline for the process of transition to detransition is also variable, with ranges varying between 4.7 to 8.5 years, which varied by sex assigned at birth and by defined transition point (eg, social, medical).^9,17,18^ These challenges highlight the need for methodological clarity. Legal gender reversal—while not synonymous with detransition, as gender identity cannot be explicitly inferred—offers a broader metric to assess stability among those who formally pursued legal gender affirmation. Legal gender change and subsequent reversal may provide insight into cases of detransition that extend beyond shifts in identity or individual feelings of regret.

The legal gender change process in Sweden is complex. According to clinical and legal praxis, an individual must receive an evaluation and official diagnosis of GD, must be prescribed gender-affirming treatment, and must be at least 18 years of age to obtain a legal gender change. Similarly, approval for legal gender reversal involves a psychological evaluation to assess the stability of one’s current gender. However, aside from age, these requirements are not strictly mandated. Therefore, an analysis of legal gender change and legal gender reversal in this context represents a unique perspective on this aspect of the detransition process, specifically as it relates to individuals who have formally sought legal gender affirmation and later reverse their legally documented gender. A previous Swedish study that examined application for legal gender changes between 1960 and 2010 showed 0.3% of applications were from individuals who sought to reverse a legal gender change, yet little is known about the most current cohort.^17^ The purpose of this study is to identify the probability of obtaining an initial legal gender change and its stability (ie, absence of legal gender reversal) in a cohort of people diagnosed with GD in Sweden between January 1, 2013, and December 31, 2023.

## Methods

### Study Design

In Sweden, a legal change of gender is documented in national administrative records; therefore, there are no missing data, and records can be combined with other national registers. We conducted a retrospective cohort study using data from the Swedish Total Population Register, the National Patient Register (NPR), and the National Cause of Death Register. This study was approved and informed consent was waived by the Central Ethical Review Board in Stockholm because of the removal of identifiable patient data. The Strengthening the Reporting of Observational Studies in Epidemiology (STROBE) reporting guideline for cohort studies was used to develop this report.

### Setting

Information was obtained on all individuals aged at least 10 years with a diagnosis of GD registered at the NPR between 2001 and 2023.^19^ The NPR consists of data on primary and secondary *International Statistical Classification of Diseases and Related Health Problems, Tenth Revision* (*ICD-10*),^20^ diagnostic codes regarding contacts with inpatient or outpatient health care. Due to the removal of sterilization requirements for legal gender change effective in 2013 and the greater completeness of the outpatient register data from the NPR (81% by 2013),^1^ data from 2013 to 2023 were used for the present study.

### Participants

Participants were included in the study if they received their first GD *ICD-10* code (ie, F64.0, F64.8, or F64.9) in the NPR between 2013 and 2023 and had not received a legal change of gender prior to their first GD diagnosis. Participants with GD-related *ICD* codes before *ICD-10* were excluded (n = 10), as were individuals with a legal gender change before their first documented GD diagnosis code (n = 46) (eFigure in Supplement 1). Participants may have received a GD diagnosis code at younger than 18 years, but a legal gender change was only accessible once participants turned 18 years of age. The terms *people with a GD diagnosis*, *assigned female sex at birth* (AFAB), and *assigned male sex at birth* (AMAB) are used throughout the present study. This is because the gender identities of participants are not recorded in the registers, and the preferred terms of the participants are unknown.

### Variables

Sociodemographic data collected for the participants included country of birth and age. Country of birth was a categorical variable with 3 levels: Sweden, another Nordic country, and outside of Nordic countries, recoded Sweden and outside of Sweden due to limited sample size in some subgroups. Age was calculated based on participants’ date of birth.

Sex assigned at birth, legal gender, and date of legal gender change were retrieved from the Swedish Population Register.^21^ Participants who changed their legal gender to the opposite of the sex that they were assigned at birth (eg, AFAB and legal gender was changed to male) were designated as obtaining a legal gender change. Participants who had a legal gender change but later reversed their legal status to the sex they were assigned at birth were designated as obtaining legal gender reversal. It is important to note that the date that a person socially transitioned or socially detransitioned cannot be inferred based on registry data.

### Data Sources and Management

Swedish national registers use the 10-digit National Registration Number, a unique personal identifier that is assigned to all Swedish residents. This personal identifier facilitates the linkage of individual records between registers. National Registration Numbers are replaced with consecutive numbers when the researchers receive data, to protect participant anonymity.

### Statistical Analysis

Descriptive characteristics of the sample and legal gender change were calculated. Kaplan-Meier estimates were used to examine the probability of legal gender change and its stability across a 10-year study period and within subgroups based on sex assigned at birth. Between-group differences were analyzed using log-rank, Mann-Whitney, and Fisher exact tests. The R statistical software environment was used for data management and analysis (version 4.3.3, Survival package 3.6.4 [R Program for Statistical Computing]).^22^ A detailed description of the statistical analysis plan can be found in the eAppendix in Supplement 1.

## Results

### Participants

A total of 7293 participants who received their first GD diagnosis between 2013 and 2023 were identified. A total of 4083 individuals were identified as AFAB (56.0%); 3210 were identified as AMAB (44.0%). One-third of the sample (2467 [33.8%]) obtained a legal gender change during the study period. Among participants who had obtained a legal gender change, the mean age at the time of the first GD diagnosis was 23.5 (IQR, 18.6-27.4) years, and 548 (22.2%) had a diagnosis before 18 years of age. The mean time from the first GD diagnosis to legal gender change was 3.2 (IQR, 2.2-4.2) years. The mean follow-up time for the sample was 4.9 (IQR 2.3-7.2) years, with a total of 36 025 person-years. A full description of the sample can be found in the Table (characteristics by sex assigned at birth are in eTable 1 in Supplement 1).

**Table.** Characteristics of People First Diagnosed With GD Between 2013 and 2023 in Swedish Register Data

Characteristic	Cohort (N = 7293)	Gender change		Legal gender reversal (n = 21 [0.3%])^a^	*P* value^b^
		No legal (n = 4826 [66.2%])	Legal (n = 2467 [33.8%])^a^		
Country of birth, No. (%)					
Sweden	6090 (83.5)	4045 (83.8)	2045 (82.9)	16 (76.2)	.39
Outside Sweden	1203 (16.5)	781 (16.2)	422 (17.1)	5 (23.8)	
Age at first GD diagnosis, mean (IQR), y	23.5 (17.8-27.4)	23.3 (17.2-27.1)	24.0 (18.6-27.4)	23.4 (19.3-27.1)	.79
Younger than 18 y at the time of diagnosis, No. (%)	NA	1939 (26.6)	548 (22.2)	2 (9.5)	.20
Age at first legal gender change, mean (IQR), y	NA	NA	27.2 (21.3-30.2)	26.1 (21-30)	.57
Age at legal gender reversal, mean (IQR), y	NA	NA	NA	29.6 (26.1-32.0)	NA
Time from first GD to legal gender change, mean (IQR), y	NA	NA	3.2 (2.2-4.2)	2.7 (1.8-3.4)	.10

Abbreviations: GD, gender dysphoria; NA, not applicable.

^a^ Includes the sample of individuals who obtained legal detransition.

^b^ Difference between legal gender change and legal gender reversal groups from Mann-Whitney and Fisher exact tests.

**Figure 1.**
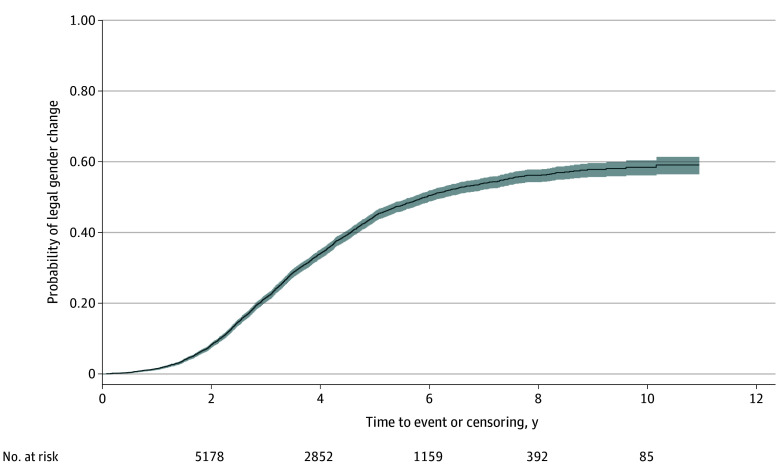
Kaplan-Meier Estimates for the Probability of Legal Gender Change for People With Gender Dysphoria Diagnosis in Sweden From 2013 to 2023 Shaded area indicates 95% CI.

The Kaplan-Meier estimate of the probability of obtaining a legal gender change was 58.3% (95% CI, 56.2%-60.4%) (Figure 1) at the end of the study period. The cumulative probability was 1.4% (95% CI, 1.1%-1.7%) for the first year, 8.1% (95% CI, 7.4%-8.7%) for the second, and 21.3% (95% CI, 20.2%-22.4%) for the third. When evaluated for differences based on sex assigned at birth, there was no statistically significant difference in the probability of obtaining a legal gender change between subgroups (AFAB, 57.9%; AMAB, 58.6%; *P* = .10) (eTable 2 in Supplement 1).

Cases of legal gender reversal were also examined. Among 2467 individuals diagnosed with GD between 2013 and 2023 who had obtained a legal change of gender, 21 cases of legal gender reversal were identified, representing 0.3% of the total sample, and 0.9% of the sample subset who had obtained an initial legal gender change (Table 1). The mean age at the time of GD diagnosis was 23.4 (IQR, 19.3-27.1) years, with 2 (9.5%; 95% CI, 1.4%-30.1%) diagnosed at younger than 18 years. The descriptive characteristics of this subgroup were similar to those of the group with legal gender change, with no statistically significant differences. In the group with legal gender reversal, the time from first GD diagnosis to legal gender change was a mean of 2.7 (IQR, 1.8-3.4) years, with no statistically significant difference from the legal gender change group (mean, 3.2 [IQR, 2.2-4.2] years) (*P* = .10). The mean age at the time of legal gender reversal was 29.6 (IQR, 26.1-32.0) years. The full sample description of participants who obtained a legal gender reversal can be found in Table 1 (stratification by sex assigned at birth in eTable 1 in Supplement 1).

**Figure 2.**
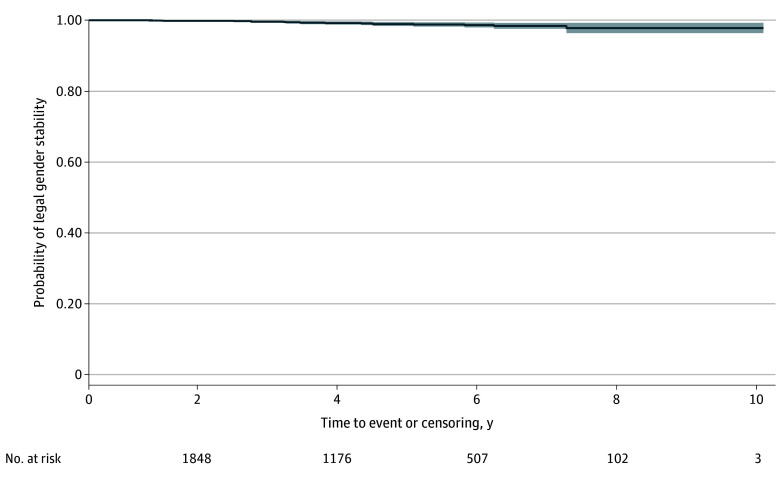
Kaplan-Meier Estimates for the Probability of Legal Gender Stability for People With Gender Dysphoria Diagnosis in Sweden From 2013 to 2023 Stability indicates lack of legal gender reversal or detransition. Shaded area indicates 95% CI.

The Kaplan-Meier estimate of the probability of legal gender change stability (ie, absence of legal gender reversal) was 97.7% (95% CI, 96.3%-99.2%) at 10 years, with considerable overlap between confidence intervals for each year (Figure 2). No statistically significant difference in the probability of legal gender reversal between AFAB and AMAB participants was observed (AFAB, 97.7%; AMAB, 97.8%; *P* = .50) (eTable 3 in Supplement 1).

## Discussion

The purpose of the present cohort study was to identify the probability of obtaining an initial legal gender change and to determine its stability (ie, absence of legal gender reversal) among people diagnosed with GD in Sweden between 2013 and 2023. After all legal gender changes in Sweden during the last decade were analyzed, the probability of a legal gender change at the end of the study period was found to be almost 60%. Unlike the current literature that predominantly reports crude prevalences, the present study uses survival analysis to report estimates of probability during a 10-year study period. This approach clarifies timing, showing the steepest increase in transitions 2 to 3 years post diagnosis, likely reflecting systemic delays in Sweden’s legal evaluation process (eg, ≥2 years for diagnosis and treatment).

However, this approach presents challenges when interpreting findings in the context of previous research. While cross-sectional studies provide estimates of 50% to 57% for legal gender changes obtained by TNB people,^23,24^ our temporal analysis contextualizes these proportions: the 58.3% probability reflects cumulative barriers, for example, Sweden’s waiting periods for assessment and legal requirements, suggesting prior estimates may underrepresent delays in accessing this form of gender affirmation. Waiting times for GD evaluation in Sweden and many other countries have increased in recent years, along with the number of people seeking health care for GD since the early 2010s.^1,17,25^ For the present cohort, the waiting and assessment times may have resulted in a process of 4 or more years for many, contributing to the observed 58.3% probability of legal gender change at the end of the 10-year study period. Therefore, this probability should be interpreted as a composite measure reflecting GD health care–related delays as well as preferences of TNB people for legal gender change.

Legal gender reversal occurred in 0.09% of our cohort who had obtained an initial legal gender change—far lower than detransition estimates in previous research (1.9% to 16%),^12,16^ which include shifts in identity, social, or medical reversal. Our analysis specifically examined solely legal gender reversals, which may reflect distinct motivations such as administrative needs as opposed to identity changes.^26^ Despite significant growth of the clinical population of individuals with a GD diagnosis in recent years and in contrast to previous studies examining surgical outcomes, prescribed hormones, or self-reported identity, the crude prevalence is on par with Sweden’s historical findings, where 0.3% of the sample had sought a legal gender reversal,^17^ suggesting legal reversal is a rare phenomenon.

These findings challenge concerns surrounding the stability of gender change, as a 97.7% probability of legal gender stability at 10 years was estimated. While some argue that late-onset GD without childhood symptoms may reflect secondary mental health or neurodevelopmental conditions instead of GD or that youths accessing gender-affirming treatments may come to regret or change their needs, no statistically significant demographic differences were observed among the cases with reversal when compared with the broader cohort. This aligns with evidence asserting that posttransition changes are often heterogeneous in their characteristics.^3,14^

### Limitations

This study has several limitations. Waiting times for initial evaluation for GD diagnosis have lengthened over the years as referrals have grown, and subsequently access to legal gender change was likely delayed. Additionally, the use of register data and subsequent application of GD diagnosis for sample inclusion also limited the understanding of participants’ gender identities to evaluate differences in legal gender change or legal gender reversal across different TNB identities. This was a significant gap, as nonbinary individuals, individuals who do not experience their gender as a masculine-feminine binary and often seek gender-affirming medical treatments, are a growing subpopulation,^27^ yet remain excluded from Swedish policies that permit only binary legal gender designations. Some studies on detransition showed that nonbinary individuals may face heightened risk due to shifts in identity or medical pathways in misalignment with their specific needs.^28,29^

While the present findings provide insights into legal gender changes and reversals, register data do not provide motivations or whether an individual would describe their experience as detransition. For some, shifts in identity and transition needs reflect an evolution of gender rather than a complete detransition.^4,26^ This measurement approach cannot be used to determine whether a legal gender reversal means that the participant is unhappy with some or all of their transition.^26^ Although legal gender reversals may indicate a misalignment with participants’ current gender identities or other changes in needs as related to the social representation of their gender,^26^ the precise reasons could not be confidently inferred, nor could we conclusively determine whether individuals reidentified with their sex assigned at birth.

The use of dates for GD diagnoses also limited our ability to establish the exact start of care. While these codes serve as an estimate of health care engagement related to GD, they do not provide the exact date of official diagnosis and onset of gender incongruence or GD, nor do they indicate when individuals began social aspects of transition or any change in care needs, including for those who obtained a legal gender reversal. Individuals who detransition through discontinuation of hormonal treatments or the reversal of another procedure, but do not seek a legal gender reversal are not represented in the current findings. This approach to proxying the phenomenon of detransition should be considered a lower-bound approximation. Therefore, these findings cannot be generalized to social detransition rates and can only be considered in the context of legal forms of detransition in a setting where nonbinary legal identification is unavailable. In future work, larger samples of individuals who obtain a legal gender reversal may provide more nuanced insight into the probability of this outcome at different time points, as in the present study the confidence intervals overlapped and caution further interpretation.

## Conclusions

This examination of legal gender change and legal gender reversal among a Swedish cohort of individuals with a GD diagnosis from 2013 to 2023 indicated that the probability of obtaining a legal gender change within 10 years of first diagnosis was almost 60%. Despite considerable growth in this population over recent years and concerns over the potential for many individuals to experience detransition, the probability of legal gender stability was approximately 98% at the end of the 10-year study period. Population trends have shown an increase in AFAB people seeking care for GD; however, no differences by sex assigned at birth for legal gender change or legal gender reversal were observed. Youths who receive gender-affirming treatment at younger than 18 years have also been cited as a concern for detransition care needs, as there has been a rise in this population accessing GD health care in recent years. This was not observed in the present data, as most individuals who legally detransitioned were in their twenties at the time of their first GD diagnosis. Further work is needed to examine whether there are stable trends in the characteristics of individuals who seek legal gender reversal over a longer period. Examining use of legal gender change can help to evaluate barriers faced by those who desire access to this aspect of gender-affirming care. Investigating legal gender reversal, or this part of a detransition process, can inform further evaluation and adaptation of the continuum of transition-related care.

## Notes

Supplement 1. **eFigure.** Participant Flowchart Based on Application of Inclusion and Exclusion Criteria **eTable 1.** Characteristics of People First Diagnosed With Gender Dysphoria Stratified by Sex Assigned at Birth **eTable 2.** Kaplan-Meier Estimates for the Probability of Obtaining a Legal Gender Change Over a 10-Year Follow-Up for People With Gender Dysphoria (GD) Diagnosis by Sex Assigned at Birth **eTable 3.** Kaplan-Meier Estimates for the Probability of Legal Gender Stability Over a 10-Year Follow-Up for People With Gender Dysphoria Diagnosis by Sex Assigned at Birth **eAppendix.** Statistical Analysis Plan

Supplement 2.
Data Sharing Statement
