# Supplementary material for: Stability After Legal Gender Change Among Adults With Gender Dysphoria
Source: JAMA Netw Open. 2025 Sep 4;8(9):e2527780. doi: 10.1001/jamanetworkopen.2025.27780 (PMC12411971; doi:10.1001/jamanetworkopen.2025.27780)
Supplement: Supplement 1. — eFigure. Participant Flowchart Based on Application of Inclusion and Exclusion Criteria eTable 1. Characteristics of People First Diagnosed With Gender Dysphoria Stratified by Sex Assigned at Birth eTable 2. Kaplan-Meier Estimates for the Probability of Obtaining a Legal Gender Change Over a 10-Year Follow-Up for People With Gender Dysphoria (GD) Diagnosis by Sex Assigned at Birth eTable 3. Kaplan-Meier Estimates for the Probability of Legal Gender Stability Over a 10-Year Follow-Up for People With Gender Dysphoria Diagnosis by Sex Assigned at Birth eAppendix. Statistical Analysis Plan [file jamanetwopen-e2527780-s001.pdf]

Supplementary Online Content

Clark KD, White R, Karamanis G, et al. Stability after legal gender change among adults with gender dysphoria. *JAMA Netw Open.* 2025;8(9):e2527780. doi:10.1001/jamanetworkopen.2025.27780

eFigure. Participant Flowchart Based on Application of Inclusion and Exclusion Criteria

eTable 1. Characteristics of People First Diagnosed With Gender Dysphoria Stratified by Sex Assigned at Birth

eTable 2. Kaplan-Meier Estimates for the Probability of Obtaining a Legal Gender Change Over a 10-Year Follow-Up for People With Gender Dysphoria (GD) Diagnosis by Sex Assigned at Birth

eTable 3. Kaplan-Meier Estimates for the Probability of Legal Gender Stability Over a 10-Year Follow-Up for People With Gender Dysphoria Diagnosis by Sex Assigned at Birth

eAppendix. Statistical Analysis Plan

This supplementary material has been provided by the authors to give readers additional information about their work.

© 2025 Clark KD et al. *JAMA Network Open*.

eFigure. Participant Flowchart Based on Application of Inclusion and Exclusion Criteria

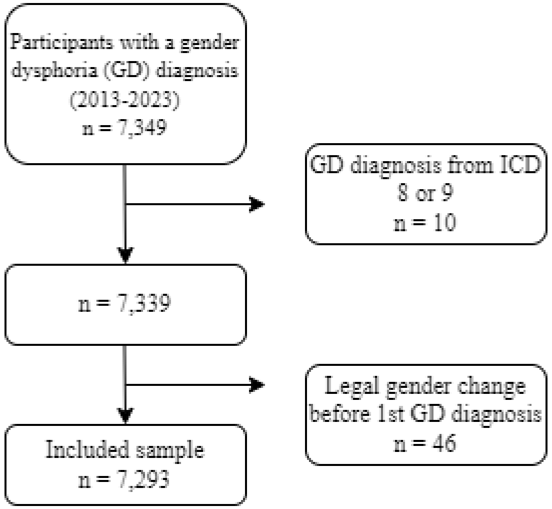

© 2025 Clark KD et al. *JAMA Network Open*.

eTable 1. Characteristics of People First Diagnosed With Gender Dysphoria Stratified by Sex Assigned at Birth

Characteristic	Cohort	No Legal Gender Change	Legal Gender Change[a]	Legal Gender Reversal
**By sex assigned at birth**				
Assigned Female Sex at Birth	4,083	2,675[a] (65·5%)	1,408[a] (34·5%)	13[a] (0·32%)
Country of Birth				
Sweden	3,536 (86·6%)	2,310 (56·6%)	1,226 (87·1%)	-[b]
Outside Sweden	547 (13·4%)	365 (8·9%)	182 (12·9%)	-[b]
Age at first GD diagnosis (M)	21·5, 7·8	21·5	21·6	24·2
Interquartile range (IQR), 25th to 75th	17 – 25	16 – 25	17 – 24	19 – 28
Age at first legal gender change (M)	*n/a*	*n/a*	25·0	26·5
IQR, 25th to 75th			20 – 28	21 – 30
Age at legal gender reversal (M)	*n/a*	*n/a*	n/a	30·2
IQR, 25th to 75th				25 – 33
Time from first GD to legal gender change (M)	*n/a*		3.3	2·4
IQR, 25th to 75th			2·3 - 4·2	1·7 – 3·4
Assigned Male Sex at Birth	3,210	2,151[a] (67·2%)	1,059[a] (33·1%)	8 [a] (0·25%)
Country of Birth				
Sweden	2,554 (79·8%)	1,735 (80·7%)	819 (77·3%)	-[b]
Outside Sweden	656 (20·4%)	415 (19·3%)	240 (22·7%)	-[b]
Age at first GD diagnosis (M)	26·0	25·5	27·1	22·3
IQR, 25th to 75th	19 – 29	18 – 29	20 – 30	20 – 22·8
Age at first legal gender change (M)	*n/a*	n/a	30·2	25·4
IQR, 25th to 75th			23 – 33	22·5 – 26·3
Age at legal gender reversal (M)	*n/a*	n/a	n/a	28·5

© 2025 Clark KD et al. *JAMA Network Open*.

IQR, 25th to 75th		26 – 29·8
Time from first GD to legal gender change (M)	3·1	3·2
IQR, 25th to 75th	2·0 – 4·0	2·8 – 3·5

[a]this group includes the sample of individuals who obtained legal gender reversal
[b]censored because of small cell sizes to protect participant anonymity
Diagnosis is between 2013-2023 in Swedish register data

© 2025 Clark KD et al. *JAMA Network Open*.

eTable 2. Kaplan-Meier Estimates for the Probability of Obtaining a Legal Gender Change Over a 10-Year Follow-Up for People With Gender Dysphoria (GD) Diagnosis by Sex Assigned at Birth

Time from First GD Diagnosis	N (at risk)	Probability (%)	95% Confidence Interval	N (at risk)	Probability (%)	95% Confidence Interval	Log-Rank Test
	Assigned Female Sex at Birth			*Assigned Male Sex at Birth*			
	4,083			3,210			$p = \cdot 1$
1 year	3,682	0·9%	0·6%-1·2%	2,792	2·1%	1·6%-2·6%	
2 years	3,019	6·9%	6·1%-7·7%	2,159	9·6%	8·4%-10·7%	
3 years	2,306	19·8%	18·4%-21·1%	1,586	23·4%	21·6%-25·1%	
4 years	1,696	32·7%	31·0%-34·4%	1,156	35·1%	33·1%-37·1%	
5 years	1,096	44·0%	42·0%-45·8%	752	44·8%	42·5%-46·9%	
6 years	672	50·3%	48·2%-52·3%	487	50·5%	48·1%-52·8%	
7 years	397	53·9%	51·7%-56·1%	321	53·6%	51·0%-56·1%	
8 years	202	55·8%	53·4%-58·1%	190	56·3%	53·5%-58·9%	
9 years	90	57·9%	55·1%-60·5%	102	57·5%	54·5%-60·3%	
10 years	40	57·9%	55·1%-60·5%	45	58·6%	55·3%-61·7%	

Data are from Sweden from 2013-2023.

© 2025 Clark KD et al. *JAMA Network Open*.

eTable 3. Kaplan-Meier Estimates for the Probability of Legal Gender Stability Over a 10-Year Follow-Up for People With Gender Dysphoria Diagnosis by Sex Assigned at Birth

Time from First Legal Gender Change	N (at risk)	Probability (%)	95% Confidence Interval	N (at risk)	Probability (%)	95% Confidence Interval	Log-rank test
	Assigned Female Sex at Birth			*Assigned Male Sex at Birth*			
	1,408			1,059			*p*=·5
1 year	1,214	99·8%	99·6%-100%	900	100%	100%-100%	
2 years	1,060	99·8%	99·6%-100%	788	99·6%	99·3%-100%	
3 years	854	99·5%	99·1%-99·9%	669	99·5%	99·1%-100%	
4 years	652	99·2%	98·7%-99·8%	524	99·0%	98·3%-99·8%	
5 years	471	98·7%	97·9%-99·5%	396	99·0%	98·3%-99·8%	
6 years	268	98·2%	97·1%-99·3%	239	99·0%	98·3%-99·8%	
7 years	104	97·7%	96·3%-99·1%	97	99·0%	98·3%-99·8%	
8 years	54	97·7%	96·3%-99·1%	48	97·8%	95·3%-100%	
9 years	14	97·7%	96·3%-99·1%	12	97·8%	95·3%-100%	
10 years	1	97·7%	96·3%-99·1%	2	97·8%	95·3%-100%	

Data are from Sweden from 2013-2023.

© 2025 Clark KD et al. *JAMA Network Open*.

eAppendix. Statistical Analysis Plan

Statistical Analysis Protocol

Introduction

While the transgender population and access to gender-affirming interventions has risen in recent decades, concerns have emerged about the potential for detransition, or the reversal of social, medical, or legal transition. Some cases of detransition have been observed, with variations in reasons and types of detransition. For example, some individuals only reverse part of their transition or pause the process. Fewer still detransition entirely, although this area of study is in its infancy. Studying detransition through the lens of legal gender changes may shed light on longer-term instances of detransition as a phenomenon, as the process of legal gender change and a subsequent reversal is complex, particularly in Sweden. The purpose of this study is thus to identify the probability of obtaining a first legal gender change and of the stability of that legal gender change (i.e., a lack of legal detransition) among people diagnosed with gender dysphoria in Sweden between 2013-2023.

Aims

Aim 1: to identify the probability of obtaining a legal gender change among people in Sweden who receive their first GD diagnosis between 2013-2023.

Aim 2: to identify the probability of legal gender stability among people diagnosed with GD following a legal gender change (Aim 1) in Sweden between 2013-2023.

© 2025 Clark KD et al. *JAMA Network Open*.

Study Design & Data Source

Swedish national registers, including those hosted by Statistics Sweden and the National Board of Health and Welfare, use the 10-digit National Registration Number, a unique personal identifier assigned to all Swedish residents. This personal identifier allows individual record linkage between registers. National Registration Numbers are replaced with consecutive numbers when the researchers receive data to protect participant anonymity. Data from the Swedish Population Register will be used to identify descriptive characteristics, sex assigned at birth, legal gender, and immigration/emigration. Data from the National Patient Register (NPR) will be used to identify the study population (i.e., people with GD). The National Cause of Death Register will be used to identify participants who may have been deceased at some point during the study follow-up period.

Study Population

Data provided by the National Board of Health and Welfare included all individuals in Sweden who, between 2001-2023, had at least one GD ICD-10 code (i.e., F64.0, F64.8, or F64.9) in the NPR. For inclusion in the present study, the first GD ICD-10 code has to occur between 2013-2023. Participants will be excluded if 1) the first ICD codes were before version 10 and 2) they had obtained a legal gender change prior to their first GD diagnosis observed in the NPR.

Variables

Primary Outcome

© 2025 Clark KD et al. *JAMA Network Open*.

Legal gender change will be determined based on the legally documented gender being different than the sex that participants were assigned at birth, as documented in the Swedish Population Register. The status of obtaining a legal gender change will be extracted with the date that it occurred.

Secondary Outcome

Legal gender reversal will be determined based on the participant obtaining an initial legal gender change (as measured in the primary outcome) and obtaining a subsequent legal gender change that matches the sex they were assigned at birth, as documented in the Swedish Population Register. The status of obtaining a legal gender reversal will be extracted with the date that the legal gender reversal occurred.

Covariates

Sociodemographic data collected for participants will include country of birth and age. Country of birth is a categorical variable provided by the Swedish Board of Health and Welfare at 3 levels: Sweden, another Nordic country, and outside of Nordic countries. The date of birth is provided by the Swedish Board of Health and Welfare as month and year. All participants will be assigned the 15th as the day of birth for analysis purposes. Age will then be calculated based on study-related events (i.e., date of first GD diagnosis, date of legal gender change, and date of legal gender reversal).

Statistical Methods

Descriptive Statistics

© 2025 Clark KD et al. *JAMA Network Open*.

Descriptive characteristics of the sample characteristics, legal gender change, and legal gender reversal will be calculated to provide summary statistics. In the event that small cell sizes are observed (<10), some descriptive characteristics may be combined.

Survival Analysis

Kaplan-Meier estimates will be used to examine the cumulative probability of legal gender change and legal gender reversal across the study period.

Handling of Missing Data

The NPR is comprised of data on primary and secondary ICD-10 diagnoses codes and from visits to hospital outpatient health care since 2001 and inpatient health care since 1965, including medical procedures (NOMESCO Classification of Surgical Procedures codes),[14] dates of admission, and discharge. The inpatient register has had nationwide coverage since 1987, while the outpatient register started with coverage between 8 and 18% during its first three years and reached 81% coverage by 2013.[1] Missing data is not a condition to be addressed with register data as events are reflected as having occurred. The absence of an event represents that the event has not occurred.

Data Analysis Plan

Primary Analysis

© 2025 Clark KD et al. *JAMA Network Open*.

The cumulative probability of legal gender change will be determined based on participants being assigned time 0 at the first date of gender dysphoria diagnosis. The event, or 1, will be the date of legal gender change. Cumulative probabilities will be determined per year (365.25 days) of follow-up, from 1 to 10 years.

Secondary Analysis

The cumulative probability of legal gender reversal will be determined based on participants being assigned time 0 at the date of the first legal gender change. The event, or 1, will be the date of legal gender reversal. Cumulative probabilities will be determined per year (365.25 days) of follow-up, from 1 to 10 years.

Subgroup Analysis

Cumulative probabilities will be analyzed among subgroups based on sex assigned at birth, i.e., assigned female sex at birth and assigned male sex at birth. Differences between groups will be tested using log-rank and chi-square tests.

Censoring/Competing Risks

The date of death, from the National Cause of Death Register, and emigration, from the National Population Register, will be used to censor data from participants following the event. Individuals who immigrate back to Sweden will be excluded after the first emigration date. In this context, competing risks are not a concern as emigration leads to solely censoring and not to an alternative event that could compete with

© 2025 Clark KD et al. *JAMA Network Open*.

death, therefore death could still occur. The process of censoring data in this manner ensures the integrity of the survival analysis and reduces bias.

Software

R version 4.3.3 with Survival package 3.6.1 will be used to conduct the study analysis.

Planned Tables and Figures

Planned Tables

Table 1 will describe the characteristics of people with GD by 1) the total cohort, 2) those who did not obtain a legal gender change, 3) those who obtained a legal gender change, and 4) those who obtained a legal gender reversal. This table will also report the characteristics of the sample within these groupings, stratified by sex assigned at birth. Follow-up time per person-years will be reported by mean, standard deviation, and interquartile ranges and by total sum. Age will be reported by mean, standard deviation, and interquartile range based on 3 time periods, 1) at the time of first GD diagnosis, 2) at the time of first legal gender change, and 3) at the time of legal gender reversal. Cells with frequencies under 10 and where categories cannot be combined will be censored to protect participant anonymity.

Table 2 will describe the results of the Kaplan-Meier cumulative probabilities for legal gender change by year of follow-up, with confidence intervals, for the total sample, and by sex assigned at birth.

© 2025 Clark KD et al. *JAMA Network Open*.

Table 3 will describe the results of the Kaplan-Meier cumulative probabilities for legal gender reversal by year of follow-up, with confidence intervals, for the total sample and by sex assigned at birth.

Planned Figures

Figure 1 will report the participants removed from the final analysis based on inclusion and exclusion criteria as a flow chart.

Figure 2 will provide a visualization of Kaplan-Meier estimates for the cumulative probability of legal gender change across the 10 years study period.

Figure 2b will provide a visualization of Kaplan-Meier estimates for the cumulative probability of legal gender change across years of follow-up, stratified by sex assigned at birth.

Figure 3a will provide a visualization of Kaplan-Meier estimates for the cumulative probability of legal gender reversal across a 10-year study period.

Figure 3b will provide a visualization of Kaplan-Meier estimates for the cumulative probability of legal gender reversal across the study period by year, stratified by sex assigned at birth.

Reporting Results

Interpreting Results

© 2025 Clark KD et al. *JAMA Network Open*.

Kaplan-Meier cumulative probabilities will be interpreted based on subtracting the probability of "survival," or not obtaining a legal gender change or legal gender reversal, from 1. The probabilities and confidence intervals will then be reported as percentages to indicate the cumulative probability of obtaining a legal gender change or legal gender reversal.

Statistical Significance

Confidence intervals will be provided, where intervals crossing 1.00 indicate no significance.

Changes in the Statistical Analysis Plan

Figure 1, and Figure 2b have been moved to a supplemental appendix. Figure 3b has been omitted due to small sample sizes and null between-group test results.

For tables 2 and 3, results by sex assigned at birth were moved to the supplemental appendix.

The outcome for aim 2 was revised to be the inverse, instead of "probability of legal gender reversal" it was revised to be "probability of legal gender stability," as reflected in the present document. This allowed the findings to be more intuitively comparable to the Aim 1 outcome and more illustrative of the intended treatment effect (survival) of legal gender change. This meant that for Aim 2, the analyses are not cumulative probabilities. Therefore, the findings were revised to be phrased as "the probability at end of the 10-year study period."

© 2025 Clark KD et al. *JAMA Network Open*.
