# Supplementary material for: Stability After Legal Gender Change Among Adults With Gender Dysphoria
Source: JAMA Netw Open. 2025 Sep 4;8(9):e2527780. doi: 10.1001/jamanetworkopen.2025.27780 (PMC12411971; doi:10.1001/jamanetworkopen.2025.27780)
Supplement: Supplement 2. — Data Sharing Statement [file jamanetwopen-e2527780-s002.pdf]

Data Sharing Statement

Clark. Stability After Legal Gender Change Among Adults With Gender Dysphoria. *JAMA Netw Open*. Published September 04, 2025. doi:10.1001/jamanetworkopen.2025.27780

Data
Data available: No

Additional Information
Explanation for why data not available: Our study includes data from Swedish National Registers, which cannot be directly shared due to confidentiality concerns and agreements. Data can be requested from the National Board of Health and Welfare in Sweden (registerservice@socialstyrelsen.se) and Statistics Sweden (https://www.scb.se/om-scb/kontakta-oss/statistikservice/fraga-oss). KDC and FP had full access to all the data in the study and take responsibility for the integrity of the data and the accuracy of the data analysis.
